# Surgical Site Infection Following the Correction of Adolescent Idiopathic Scoliosis With ApiFix: A Retrospective Study Analyzing Its Incidence and Recurrence

**DOI:** 10.7759/cureus.34494

**Published:** 2023-02-01

**Authors:** Konstantinos Zygogiannis, Eleni Pappa, Spyridon I Antonopoulos, Georgios Tsalimas, Konstantinos Manolakos, Ioannis Chatzikomninos, Savvas Moschos, Georgios C Thivaios, Dimitrios Kalatzis, Anastasios Kalampokis

**Affiliations:** 1 Trauma and Orthopaedics, Laiko General Hospital of Athens, Athens, GRC; 2 5th Orthopaedic Department, KAT General Hospital, Athens, GRC; 3 Orthopaedics, KAT General Hospital Attica, Κηφισιά, GRC; 4 6th Orthopaedic Department, Kat General Hospital, Athens, GRC; 5 Spine and Scoliosis Department, KAT General Hospital, Athens, GRC; 6 Spine Department, KAT General Hospital, Athens, GRC; 7 Orthopaedics and Traumatology, Laiko General Hospital of Athens, Athens, GRC; 8 Spine Department, Kat General Hospital, Athens, GRC

**Keywords:** urgent debridement, scoliosis surgery complications, early postoperative complication, postoperative wound infection, adolescent idiopathic scoliosis (ais)

## Abstract

Background and objective

ApiFix (OrthoPediatrics, Warsaw, IN) is an internal brace used for the correction of adolescent idiopathic scoliosis (AIS) classified as Lenke 1 or 5 with a Cobb angle of 35-60 degrees that decreases to ≤30 degrees on lateral side-bending radiographs. Since the indications are very specific, it is not a common procedure. Our study aimed to evaluate the incidence of surgical site infection (SSI) and its recurrence following treatment with ApiFix.

Materials and methods

A retrospective study of 44 cases of AIS treated at our center from 2016 to 2022 with ApifiX was conducted. Two patients who presented with SSI were initially treated with irrigation and debridement (I&D) following antibiotic therapy.

Results

A total of 44 patients with a mean age of 15.1 years were evaluated. Two of our patients presented with early-onset infection while one of them presented after the end of treatment with a skin ulcer due to septic screw loosening. The removal of the ApiFix implant revealed a pedicle abscess during the screw removal.

Conclusions

In this study of 44 patients, we observed two cases of infection and one case of reinfection. Given the limited muscle detachment and short operating time needed for Apifix, statistics suggest that the risk of SSI is always present. Further randomized trials are needed to gather more evidence on this subject.

## Introduction

Adolescent idiopathic scoliosis (AIS) is a condition that affects 1-3% of individuals aged between 10 and 16 years [1]. It is characterized by a structural lateral curvature of the spine with a significant rotatory component. Adolescents with curves larger than 40-45° are candidates for surgical management of the spine curvature, which includes the spinal instrumentation and posterior fusion of 10-12 vertebrae. However, there is a need for an intermediate surgical intervention to deal with mild to moderate AIS curves with a less invasive and less extensive surgical field, which will be able to address the correction of the aforementioned deformity [2,3].

The ApiFix device (OrthoPediatrics, Warsaw, IN) is implanted in a less invasive fashion and with fewer instrumented segments, playing the role of an “internal brace”. Moreover, after the installation of the ApiFix system, the patient is likely to be mobilized right postoperatively, which also gradually corrects the spinal deformity by rod elongation. During surgery, the initial distraction enables some correction of the deformity. However, no fusion is performed [4].

However, the surgical correction of AIS is associated with certain postoperative complications. More specifically, the infection of the surgical site, either superficial or deep, after scoliosis is a commonly reported complication. According to the latest Scoliosis Research Society (SRS) report, the infection rates following the correction of AIS range from 0.9 to 3% [3]. In the current literature, the adverse effects of infection on the clinical results of surgical correction of AIS have been examined; the infection might lead to instrumentation removal and loss of the postoperative curvature correction and functional results, in comparison with the non-infected patients. According to several case series, the rates of early and delayed postoperative infection after the surgical correction of spinal deformity in AIS usually range from 1.4 to 6.9% [5]. Nonetheless, these studies are single-center studies, with some of them performed by a single surgeon.

The objective of this study was to determine the prevalence and recurrence of surgical site infection (SSI) following surgery for AIS with ApiFix. To the best of our knowledge, this is the first study to investigate this particular topic.

## Materials and methods

We retrospectively analyzed 44 patients who underwent surgical treatment of idiopathic scoliosis with an ApiFix device at a single spine center under three different surgeons between the years 2016 and 2022. Adolescents with congenital or neuromuscular scoliosis were excluded as there is no surgical indication of the use of the ApiFix device in such cases. The inclusion criteria were scoliosis of Lenke 1A type and patients with a minimum of one year of radiological and physical follow-up. The basic procedures and steps of maintaining asepsis were the same for all patients. The pre-surgical chemoprophylaxis protocol included coverage with vancomycin 1 g every 12 hours for 24 hours postoperatively. Ethical approval was obtained from both the scientific committee and the spinal surgery unit at the hospital where the study took place. Furthermore, a consent form was signed by all the participants in the study.

Medical records including the details of the initial surgery, clinical symptoms, laboratory and microbial culture results, and treatment strategies were obtained. Preoperative radiology imaging, including standing posteroanterior (PA) and lateral radiographs, right and left supine bending X-rays, as well as the most recent postoperative radiographic examination of all study patients were examined. Furthermore, the Lenke Classification system was used for the categorization of the curve pattern of each patient, by using the Cobb method.

The SRS outcome survey was completed by all the included patients, either during their most recent follow-up visit or remotely. The IBM SPSS Statistics v12.0.1 (IBM Corp., Armonk, NY) was used for the analysis of the results, including descriptive statistics such as frequencies for both categorical and ordinal variables, and means, medians, standard deviations (SD), and ranges for continuous variables. Moreover, univariate analyses were performed using independent t-tests. A p-value ≤0.05 was considered statistically significant.

## Results

Out of the 44 patients analyzed, excluding a history of prior spine surgery and curves of non-idiopathic scoliosis, three patients (6.81%) with a median age of 14.8 years developed SSI, with a recurrence observed in one patient (2.27%). Because of the difficulty of classifying an infection as superficial or deep, all SSIs that met the CDC criteria were grouped together. The initial course of therapy included IV antibiotics following oral administration and dressing changes. Moreover, two of our patients needed irrigation and debridement (I&D) while instrumentation removal for one case was necessary due to recurrent infection and clinical deterioration leading to sepsis. Curiously, after the removal of ApiFix due to treatment completion, removing one screw resulted in pus leakage. Two cultures revealed *Staphylococcus capitis* and the third showed *Staphylococcus epidermidis*. Among the studied antibiotics, the sensitivity pattern revealed a good response to vancomycin, which comprised the main course of IV treatment including rifampicin. The mean weight of patients who did not develop SSI was 51.9 kg while that of patients who developed postop infection was 55.2 kg. The following factors were examined as potential risks: patient weight (kg) and blood loss (mL) during surgery and the first two days postoperatively. For every extra level fused, the risk of SSI occurrence increased by 14%, and for every 1 kg of extra body weight, the risk increased by 4%. There was no evident correlation between the appearance of SSI and other measured variables (surgical exposure, surgeon, gender, rod material, preoperative Cobb angle) (Tables 1, 2).

**Table 1:** Treatment and outcomes

Treatment	Number of patients	Outcomes
Oral antibiotics, dressing changes	1	Without sequelae, superficial wound infection with secondary skin healing
Irrigation and debridement, instrumentation removal, and antibiotics (symptomatic)	1	With sequelae, the final treatment included implant removal due to clinical deterioration of the patient and early recurrence of the infection
Irrigation and debridement and IV oral antibiotics (completion of therapy, ulcer due to septic screw loosening)	1	Without sequelae, the removal of the ApiFix device after therapy completion revealed a pedicle abscess during screw removal

**Table 2:** Statistical analysis

Variables measured	P-value	Mean value
Weight (kg)	0.002	51.9
Number of fused levels	0.013	4.8
Gender	0.231	Female
Surgeon	0.867	3
Preoperative Cobb angle	0.492	43.6
Rod material	0.877	Titanium alloy device

## Discussion

SSI following pediatric scoliosis surgery is a rare but devastating complication that can have either an early- or late-onset appearance [6]. Typically, such pathologies require surgical debridement and prolonged hospitalization for intravenous antibiotic therapy. In some cases where the infection is classified as superficial, secondary healing may be acceptable. Recurrent SSI may exhibit a strong indication for implant removal. ApiFix procedure is considered minimally invasive since muscle detachment is unilateral, requiring less time for surgical exposure, with low-profile screw insertion.

Carreon et al., in a prospective cohort study of 702 patients who had undergone surgical correction of AIS, reported that the prevalence of non-neurologic complications was 15.4%, and five of these cases (0.71%) presented with wound infection [7]. Factors associated with increased risk of SSI are the history of renal pathologies, increased intraoperative hemorrhage, and prolonged posterior surgical and anesthesia time [8]. The prevalence in this study is significantly lower compared to Marks et al.'s study where the sample size was almost double. However, between those studies, the surgical approaches and the number of spine centers participating were totally different. More severe curve patterns often require extended muscle and bone detachment, combined approaches, and involve extended blood loss, and considerable intraoperative spine manipulation. As a result, even if the prevalence of infection in some studies may differ, the severity of curve patterns may equally contribute indirectly. In ApiFix cases, the surgical time is significantly less in comparison with the classic instrumented posterior fixation; soft tissue dissection is unilateral and blood loss is minimal with children usually discharged after 24 hours of hospitalization.

Marks et al., reviewing 1757 patients in a multicenter prospective study, found that 28 of the patients developed SSI (1.6%) within the first 90 days postoperatively [1]. The only strong correlating factors mentioned were the amount of blood loss and the fusion levels. Some elements that are often overlooked, such as the cleaning procedure of the skin [9], operating room trafficking [10], use of wound drains, surgical team decolonization [11], and operating room habits, may contribute to the occurrence of surgical site infection. Yet, those factors were not mentioned in the database. The combined anterior and posterior approach in demanding cases seems to be associated with such complications.

Rihn et al. have reported that the most common cause of infection included skin flora such as *Staphylococcus epidermidis*, suggesting that contamination induced by skin flora leads to a longer incubation period due to the low virulence of these organisms [12]. A plethora of literature mentions plans of action to reduce the incidence of infection, such as chlorhexidine pre-wash [9], alternative options of skin closure with antimicrobial sutures, preoperative nutritional assessment, and vancomycin powder [13]. In our study, only three patients presented with Staphylococcus SSI, and statistical significance in the setting of small sample size may not be relevant. The fact that muscle detachment and blood loss were minimal in all cases indicates that all factors can contribute equally to the occurrence of such complications.

## Conclusions

SSI, especially after AIS correction, remains a troublesome entity even in the most experienced hands. The statistical significance found in a small sample size and the fact that all patients were from a single spine center were limiting factors of this study. SSI may prove devastating for children since prolonged hospitalization may often be necessary. A strict surgical protocol of surgical asepsis and postoperative dressing changes may contribute to the prevention of contamination with pathogens. This study suggests that even in minimally invasive surgical procedures involving healthy patients, there is always the risk of infection when an orthopedic implant is involved.
